# Privacy-preserving storage of sequenced genomic data

**DOI:** 10.1186/s12864-021-07996-2

**Published:** 2021-10-02

**Authors:** Rastislav Hekel, Jaroslav Budis, Marcel Kucharik, Jan Radvanszky, Zuzana Pös, Tomas Szemes

**Affiliations:** 1 Geneton s.r.o, Bratislava, Slovakia; 2 Faculty of Natural Sciences, Comenius University, Bratislava, Slovakia; 3 Slovak Centre of Scientific and Technical Information, Bratislava, Slovakia; 4 Comenius University Science Park, Bratislava, Slovakia; 5 Biomedical Research Centre, Institute of Clinical and Translational Research, Slovak Academy of Sciences, Bratislava, Slovakia

**Keywords:** Genomic privacy, Personal data, Genomic reads

## Abstract

**Background:**

The current and future applications of genomic data may raise ethical and privacy concerns. Processing and storing of this data introduce a risk of abuse by potential offenders since the human genome contains sensitive personal information. For this reason, we have developed a privacy-preserving method, named Varlock providing secure storage of sequenced genomic data. We used a public set of population allele frequencies to mask the personal alleles detected in genomic reads. Each personal allele described by the public set is masked by a randomly selected population allele with respect to its frequency. Masked alleles are preserved in an encrypted confidential file that can be shared in whole or in part using public-key cryptography.

**Results:**

Our method masked the personal variants and introduced new variants detected in a personal masked genome. Alternative alleles with lower population frequency were masked and introduced more often. We performed a joint PCA analysis of personal and masked VCFs, showing that the VCFs between the two groups cannot be trivially mapped. Moreover, the method is reversible and personal alleles in specific genomic regions can be unmasked on demand.

**Conclusion:**

Our method masks personal alleles within genomic reads while preserving valuable non-sensitive properties of sequenced DNA fragments for further research. Personal alleles in the desired genomic regions may be restored and shared with patients, clinics, and researchers. We suggest that the method can provide an additional security layer for storing and sharing of the raw aligned reads.

**Supplementary Information:**

The online version contains supplementary material available at 10.1186/s12864-021-07996-2.

## Background

The advancements in DNA sequencing technology support increasingly complex and accurate interpretation of genomic data which leads to exposure of sensitive personal information [7, 11, 30, 32]. Genomic privacy of an individual may be breached through different types of attacks, in particular, identity tracing, attribute disclosure, and completion attacks [21]. To address this issue, genomic data is regulated as personal data [29, 31] and must be protected accordingly. On the other hand, it is important to support availability of and access to genomic data for precision medicine, genomic research, forensic investigation, and recreational genomics [2, 23].

In general, many genomic analyses are focused on short genomic variants and a typical method of the prior art extracts these variants from the underlying genomic reads. Such method stores only the variants in a secure form and discards the original genomic reads or encrypts them, so they can be reanalysed in future [3, 4, 10, 13, 17, 33]. However, it is a common practice to confirm uncertain variants by manual examination of the underlying mapped genomic reads (alignments), and specific variants can remain undetected due to their misclassification as sequencing errors [16]. Moreover, the current variant calling algorithms are not mature, and it is unknown which type of data produced by the sequencing process will be necessary for any future algorithms [5]. Alignments carry additional information which can be employed directly in the detection of structural variations such as copy number variations (CNVs) or aneuploidies in clinical non-invasive prenatal testing (NIPT) [20, 25, 26]. The detection methods for these variants do not consider short variations and require coverage data provided by alignments.

Various privacy-preserving solutions for processing genomic data have been proposed. Lauter et al. adapt several algorithms used in genome-wide association studies (GWAS) to process genomic data encrypted with homomorphic encryption [17]. Sousa et al. use this type of encryption to securely store and search encoded variants on a cloud server [33]. A secure multiparty computation can ensure diagnosis of causal variants in a group of patients affected by the same Mendelian disorder [13]. To the best of our knowledge, only few privacy-preserving methods for genomic reads exist. Among them, Decouchant et al. [9] use Bloom filter to classify unaligned genomic reads to privacy-sensitive or non-sensitive, improving the same previous approach for short reads [8]. The protocol proposed by Ayday et al. [5] encrypts genomic reads and stores them in a biobank, from which a trusted medical unit can request a range of nucleotides without revealing the range to the biobank. Huang et al. [12] presented a novel file format as an alternative to BAM and CRAM files, offering compression and encryption of aligned reads and their selective retrieval.

In this paper, we present our methodology which preserves raw alignments and their unique properties without disclosing personal information while facilitating secure storage of alignment data with the support of dynamic consent approach. More specifically, we mask personal single nucleotide variation (SNV) alleles within alignments of a sequenced genome while preserving existing alignment data (coverage, quality, etc.). The masking solution is reversible, allowing any user with access to masked personal alleles to unmask them within any arbitrary region of the genome. The user can also share access to a subset of the masked alleles in encrypted form with another user. Thus, a patient could share the subset of genuine reads related to a particular gene with a medical unit. We implemented the proposed methods, validated them with real personal genomic data, and evaluated the reported genomic variation.

## Results

We validated the performance of our methodology with five distinct analyses: (1) the single case study on a selected personal genomic sample, (2) the principal component analysis (PCA) on a set of genomic samples to show the effect of masking on personal alleles, (3) the second PCA analysis on two distinct populations, (4) the comparison of VCF files called on original samples with VCF files called on masked samples, and (5) the comparison between detected pathogenic variants in clinically relevant genes before and after masking.

### Single case study

**Fig. 1:**
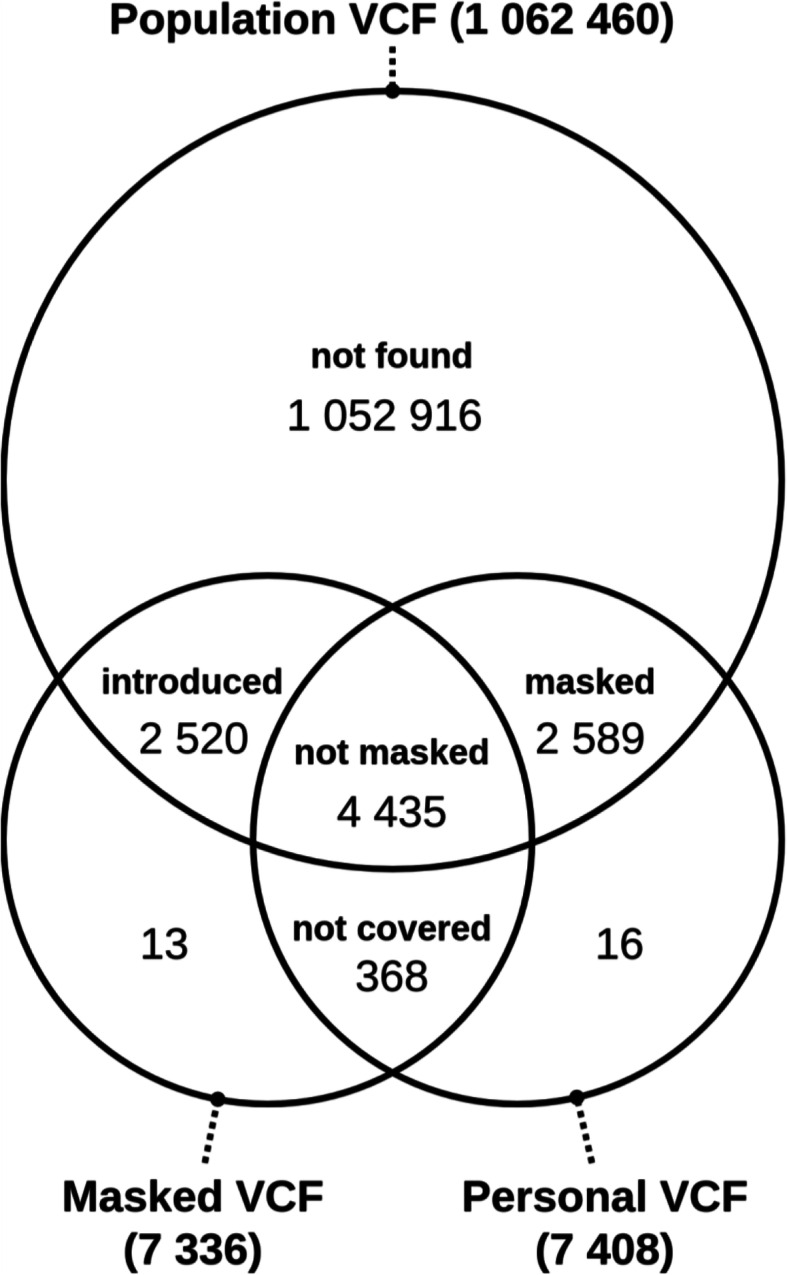
Intersections between the sets of positions with alternative alleles from three VCF files: population VCF, personal VCF, and masked VCF

The performance of the masking method was evaluated by a comparison between (1) called variants on a BAM file with personal genome from central European population, (2) called variants on the corresponding masked BAM file, and (3) the set of variants from the non-Finnish European population in The Genome Aggregation Database (gnomAD) [15] database. We selected only the passing variants with total coverage and quality above 30 from both personal and masked VCF files to provide high-confidence results. We identified five categories of variant positions from personal VCF, masked VCF, and population VCF (Fig. 1). (1) *Not found*: A vast majority of variant positions in the population VCF is not found in the personal VCF. This was expected as the population VCF is called on thousands of personal genomes, and masking of rare variants tends to result in a homozygous reference. (2) *Masked***:** This case occurs when a homozygous reference allele masks a homozygous alternative allele. (3) *Not masked*: An alternative allele at this position was either preserved or replaced by another alternative allele while zygosity may be changed. (4) *Introduced***:** When an alternative allele replaces a reference allele at a homozygous position, a new variant appears. (5) *Not covered*: A set of personal variant positions not covered by the population VCF. These are presumably rare variants or variants specific for a particular local population that were not present in the gnomAD database.

**Fig. 2:**
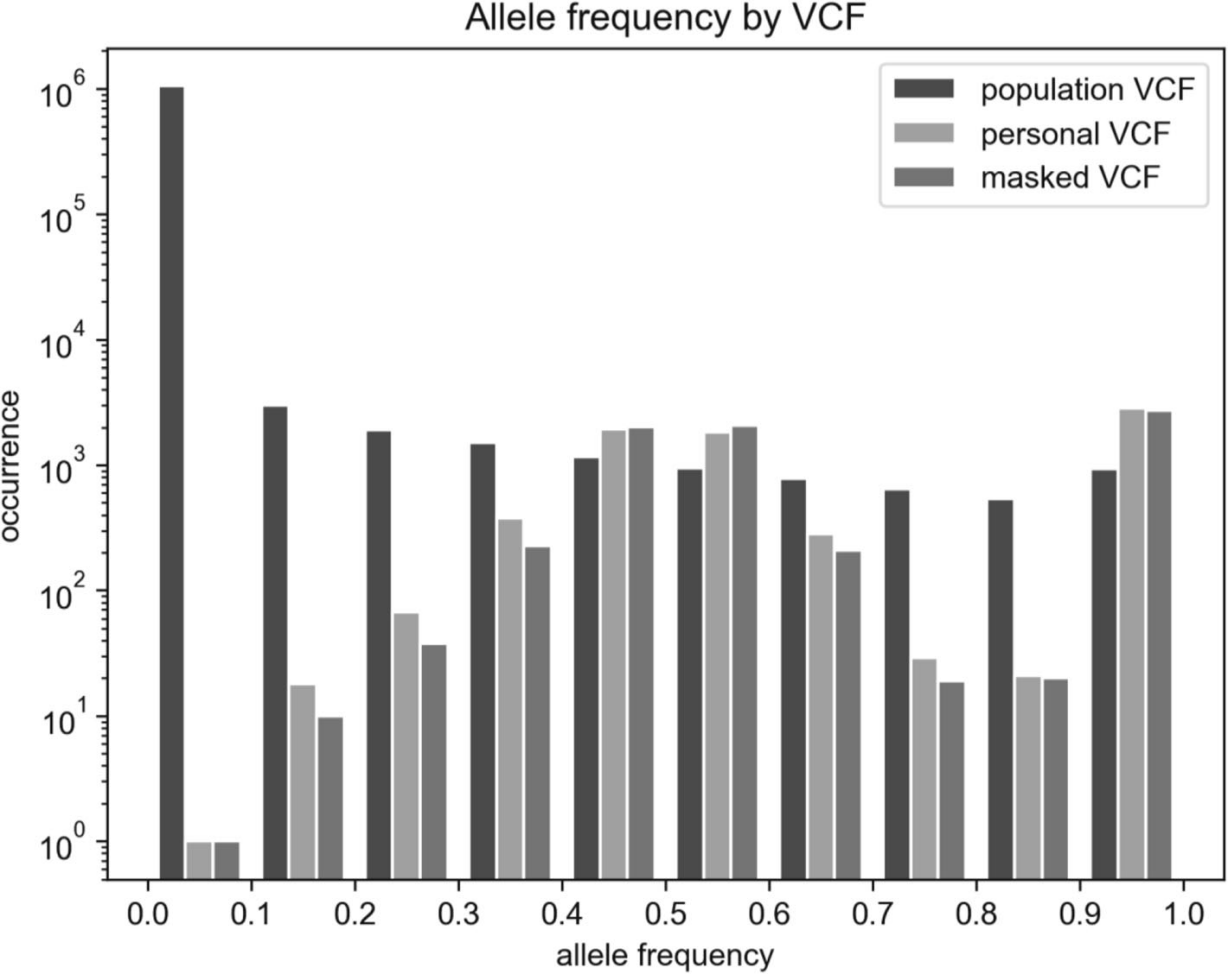
The distribution of alternative allele frequency reported by population VCF, personal VCF, and masked VCF

We compared the distributions of alternative allele frequencies by VCF to show their nature and the effect of masking (Fig. 2). The population VCF contains a vast amount of low-frequency alleles which have little chance to be introduced by the masking process into the masked VCF despite considering every variant covered by personal BAM. In case of the personal VCF, personal allele frequency has the anticipated ratio of 0.5 for a heterozygote and 1.0 for a homozygote. However, actual ratios may vary due to low coverage or sequencing errors. As can be observed, masked VCF preserves the distribution of personal allele frequency to a considerable extent.

**Fig. 3:**
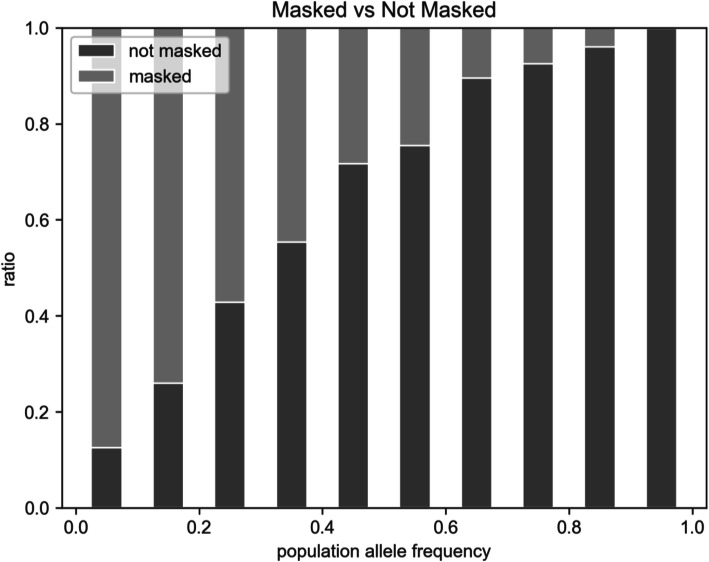
The ratio of masked to not masked alleles and its relation to population allele frequency

Furthermore, we compared the distribution of alternative population allele frequencies between the masked VCF and the not masked VCF (Fig. 3). The ratio of masked alleles increases with decreasing allele frequency, therefore, rare variants have a higher chance to be masked by the method. Similarly, the ratio of introduced alleles increases with decreasing allele frequency. On the other hand, common population alleles have a lower chance to be masked or introduced; nonetheless, they are specific for the population and not for an individual.

Finally, we compared the alleles within the not masked set between the personal VCF and the masked VCF. The alternative alleles from both VCFs were joined by their positions, allowing a direct comparison of an alternative allele and its frequency between the two files. An alternative allele was replaced by another alternative allele in only 14 (0.32%) from the total of 4435 reported positions making this issue negligible. We compared frequencies of the 4421 remaining positions with matching alleles between the personal and masked file and found a mismatch in 1515 (34.27%) of them. The changes of frequencies of alternative alleles in these positions were caused by a change of a homozygous pair of alleles to a heterozygous one or vice-versa due to the masking method.

### The masking effect on personal alleles

In the first PCA masking analysis, we merged all the passing SNVs from both personal and masked VCFs (66 files in total) into a single VCF. The PCA analysis was performed on this file using PLINK [27], which is a toolset for genome association analysis. The analysis was performed twice, each time with a different set of population frequencies (VOF file). Firstly, with all populations from gnomAD database, and secondly, with the non-Finnish European population as it best matches the central European population of locally-sequenced individuals [6].

**Fig. 4:**
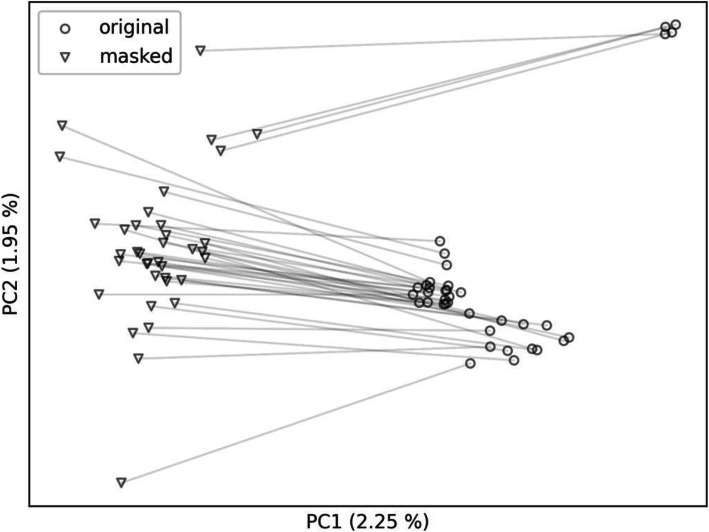
Personal VCFs are clearly shifted from the original local population (non-Finnish European) to VCFs masked with alleles from all gnomAD populations. Lines link the individual original BAMs (circles) with their masked counterparts (triangles)

**Fig. 5:**
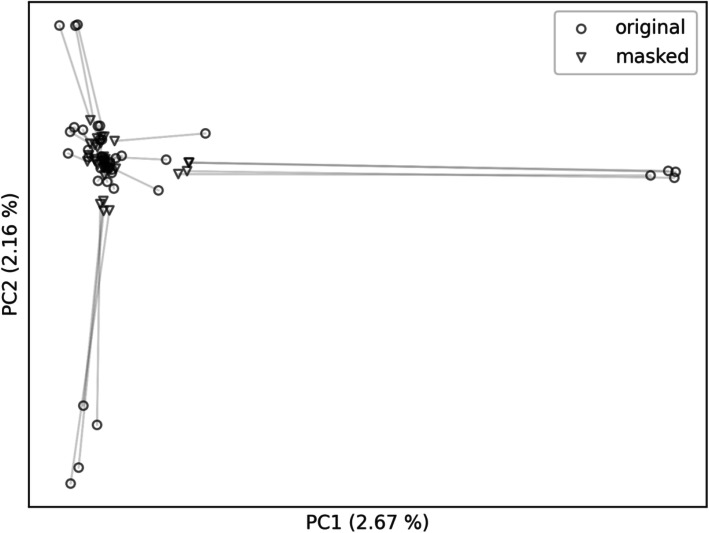
All masked VCFs, including outliers in their personal form, are clustered in the same region. The lines link the individual original BAMs (circles) with their masked counterparts (triangles). For details of the cluster, see Fig. 6

**Fig. 6:**
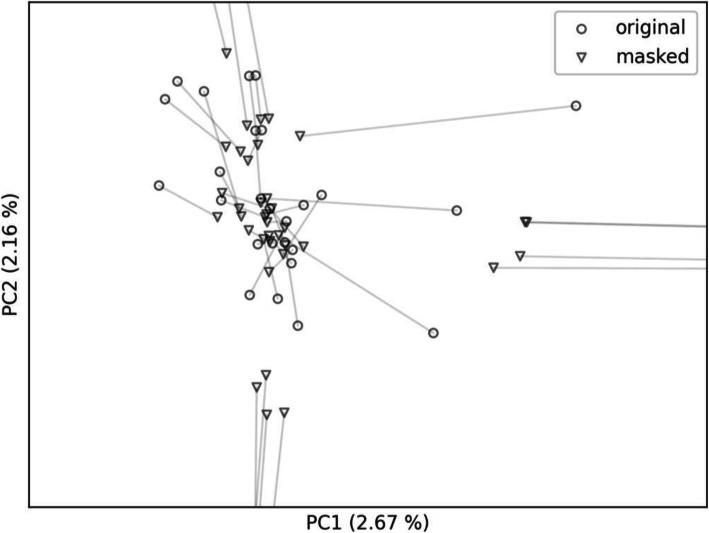
The detail of the cluster from Fig. 5. The lines link the individual original BAMs (circles) with their masked counterparts (triangles)

We plotted the first two principal components and distinguished the original and masked VCFs with a marker type. In the first case (Fig. 4) we masked the VCFs using the whole gnomAD variation as population allele frequencies. As a result, the masked VCFs are clearly separated from the personal VCFs as two different clusters, implying that the masking using whole gnomAD variation caused a shift from the population of origin to the mixture of gnomAD populations. In the second case (Figs. 5 and 6), we selected only the non-Finnish European gnomAD population allele frequencies thus creating a single cluster. This time, the masked VCFs cannot be unambiguously mapped to corresponding personal VCFs since they stay within the same population space. Moreover, outliers – the VCFs with specific genotypes – are shifted towards the population cluster.

#### The masking effect on personal alleles from two distinct populations

**Fig. 7:**
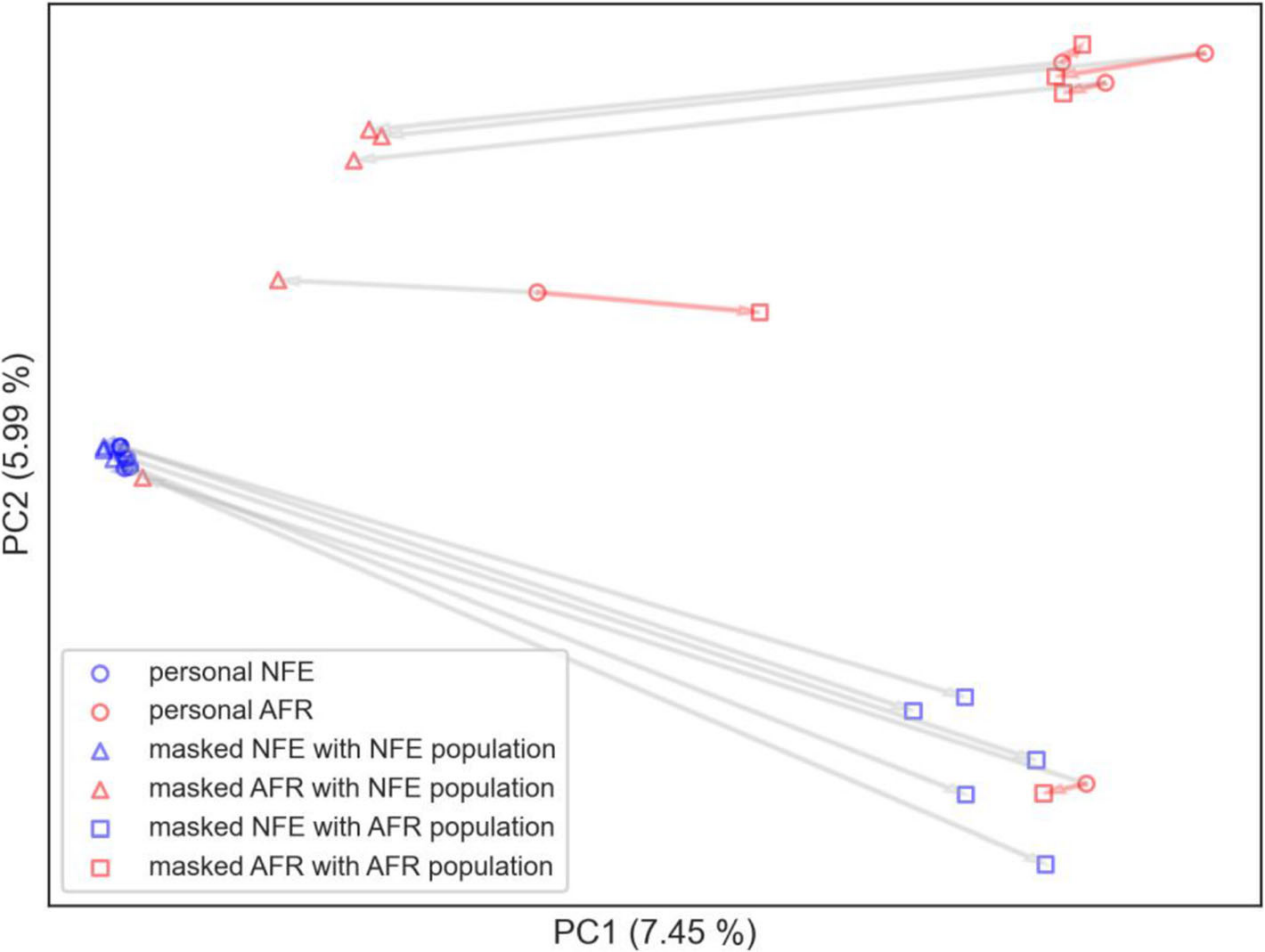
Personal and masked VCFs from African (red) and non-Finnish European (blue) population. Each of the two personal VCFs was masked with non-Finnish European (triangle) and African (square) population allele frequencies. The arrows point to a position of the masked version of the personal VCF, while the coloured arrow denotes masking with population allele frequencies matching the origin of the personal VCF

In the second PCA analysis, we compared the masking effect jointly on exome samples from the 1000 Genome Project [1]. To find and plot the principal components, we used the same approach as in the first analysis. In particular, we selected five samples from the African population (AFR) and five samples from the non-Finnish European population (NFE). We masked each sample with both the AFR and the NFE allele frequencies from the gnomAD database. Personal samples from the NFE population are clearly clustered on the left, while personal samples from the AFR population are mostly separated and placed on the right side of the image (Fig. 7). The samples masked with the matching population stay in the vicinity of the related personal samples, i.e. they cannot be distinguished from other samples within this population. In contrast, samples masked with the other population are shifted towards this population area but may not reach full similarity with this population. Nevertheless, this approach masked the original population of the sample. In conclusion, the masking of personal variation is effective when the population of masking frequencies matches the population of the personal sample. We provide additional analyses confirming this statement in the Additional file 1; Section 4; Figs. 2, 3, 4, 5.

### Masking of pathogenic variation

In this section, we focused the analysis on clinically relevant variants which are considered to be privacy-sensitive. More specifically, this concerns the variants classified as pathogenic or likely pathogenic based on the current guidelines on the interpretation of sequence variants [28]. We manually examined the pathogenic variants according to the annotations provided by the Ensembl Variant Effect Predictor [19] within the clinically relevant genes defined by American College of Medical Genetics (ACMG) [14] in all 33 personal and masked samples. Three variants from the personal set were masked, and three new variants were introduced in the masked set. A single variant had no population allele frequency defined and could not therefore be processed by the method. Within the personal set, we found 5 different variants across 12 samples. One of them (rs1805124) was present in 10 samples. Within the masked set, we found 6 different variants in 19 samples while one of them (rs1805124) was present in 16 samples. The population allele frequency of the rs1805124 variant was 23.96% which resulted in low masking probability and high introduction probability. We also observed a zygosity change in this particular variation in a single masked sample. This analysis demonstrated the effectiveness of masking personal alleles, especially those with low population frequency. Nevertheless, clinically relevant alleles may be missing from public variation archives and the allele population frequencies must be known beforehand in order to mask the related variants.

### Reversibility of masking

We used 33 clinical exomes to validate the reversibility of the masking method. First, we masked and unmasked all the 33 samples. Next, we called variants on both original and masked samples, producing two sets of 33 VCF files. We compared the number of called variants for each sample between the two sets and also their file contents with the standard Linux shell diff command. The comparison showed that the unmasking method fully restored the original alignment data, resulting in two identical call sets for each genomic sample, containing 276,295 variants in total. Moreover, we confirmed an exact match of records from each sample between its original and unmasked version in both BAM and VCF files.

## Discussion

The growing number of sequenced genomes and improving genomic interpretation makes their carriers and their relatives vulnerable to privacy violations [7, 11, 21, 31]. It is therefore essential to prevent unauthorised copying, modifying, and sharing of private genomic data. On the other hand, sharing of data is a fundamental part of genomic research inevitable in clinical practice [23, 32]. Given these points, a practical solution to genomic privacy represents a certain trade-off between privacy and utility of the data [21, 31].

Many methods for preserving genomic privacy encrypt genomic data entirely aiming to secure personal variants [3, 4, 10, 13, 17, 33]. For example, the encryption keys are in the possession of a manager which does not require the participation of a patient, except for his or her consent [5]. Although the protocol distributes the roles in secure processing of genomic data to distinct parties, it relies on a trusted medical unit, which possesses individual access rights to different parts of the data. On the contrary, our method allows an individual to retain full control over his or her digital genome, supporting a dynamic consent approach with limited access only to his or her personal alleles.

Other methods detect sensitive personal reads using the Bloom filter, which is built from a public database of genomic variation and relies on exact matching through hashing [8, 9]. However, the exact mechanism for obfuscation of the sensitive reads is not further explained. The devised format for secure storage of compressed aligned reads in another paper creates a substantial overhead for downstream analyses, since standard bioinformatic tools do not support it [12]. This format provides a solution to privacy control of genomic reads; however, it does not address the issue of secure sharing of retrieved reads.

We have demonstrated that using allele frequencies from the same population of origin as the masked sample makes the masking more effective. Specifically, the set of masked variants resembles a generic sample from this population and cannot be trivially mapped back to the original set in a pool of similar samples. However, considering that masking population allele frequencies are public, the offender can always tell which genomic positions may be masked, and exploit any rare personal variants not covered by the method. This could be mitigated by using a more comprehensive set of masking population allele frequencies as well as by the addition of random masking (Additional file 1; Section 3). In future research, we intend to mask all personal variants not covered by population allele frequencies using called variants on personal BAM as yet another input to the methodology. Furthermore, the method can be instantly improved by masking indels and short tandem repeats using the presented approach for masking SNVs when comprehensible population frequencies for these types of variation become available.

## Conclusions

Our method masked SNV alleles within genomic alignments and securely preserved them using standard RSA encryption. We were also able to restore original alignments using the encrypted masked alleles with the associated access key or provide partial access to these alleles to another entity without disclosing them to a third party. A masked BAM preserves natural population distribution of alternative allele frequencies, which may be an advantage against a potential offender, since he or she cannot tell if the BAM is masked. The extent of masking depends on the comprehensiveness of the population allele frequencies used as an input. It can therefore be continually improved with expanding catalogues of genomic human variation. Its effectiveness depends on proper selection of the masking population. For example, using masking allele frequencies from the same population as the sample is suitable for hiding an individual within this particular population. We can also use masking allele frequencies from a different population to make the encrypted sample resemble a sample from this particular population. In some cases, personal re-identification from the masked genome is still possible despite masking of the SNVs. Regardless of the above, the purpose of Varlock is not de-identification of a genome, or replacement of standard security methods. Instead, we believe that concepts presented herein will find application in future medical and laboratory information management systems.

## Methods

**Fig. 8:**
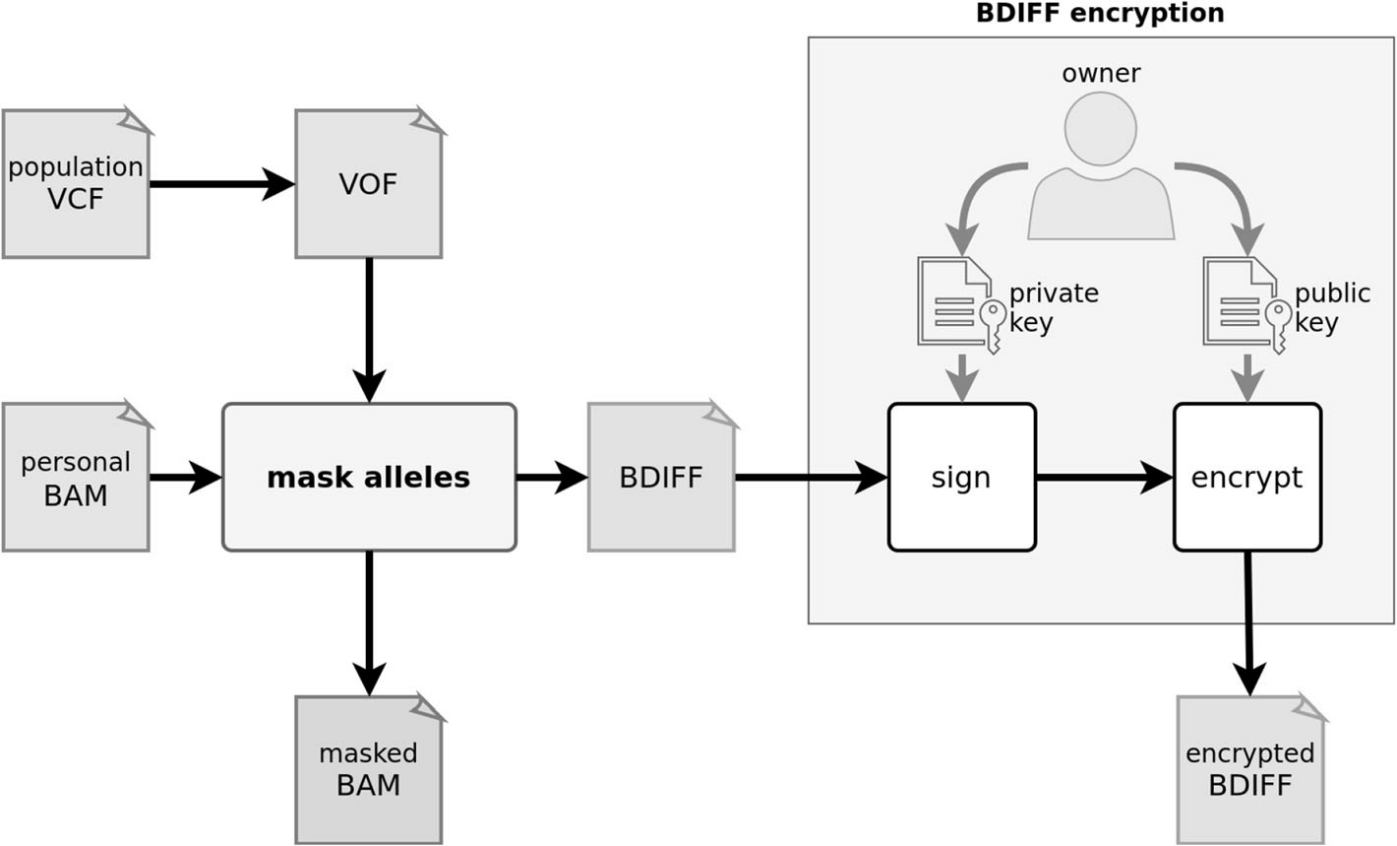
Workflow of the masking method, where BAM file and VOF file are processed into the masked BAM and BDIFF files. The BDIFF file is subsequently encrypted

The Varlock methodology provides methods for masking, unmasking, and sharing of personal alleles found in alignments stored as a BAM file. More specifically, the masking method (Fig. 8) masks personal alleles found in alignments using publicly known population allele frequencies from a dedicated VOF file (Additional file 1; Section 1; Tables 1 and 2). The output set of masked alleles represents all differences between original and masked alignments and is stored in a dedicated BDIFF file (Additional file 1; Section 2, Tables 3 and 4). The masked alleles are encrypted as a single file using an asymmetric encryption scheme (Additional file 1; Section 2; Fig. 1) only enabling the holder of the associated private key to decrypt them.

**Fig. 9:**
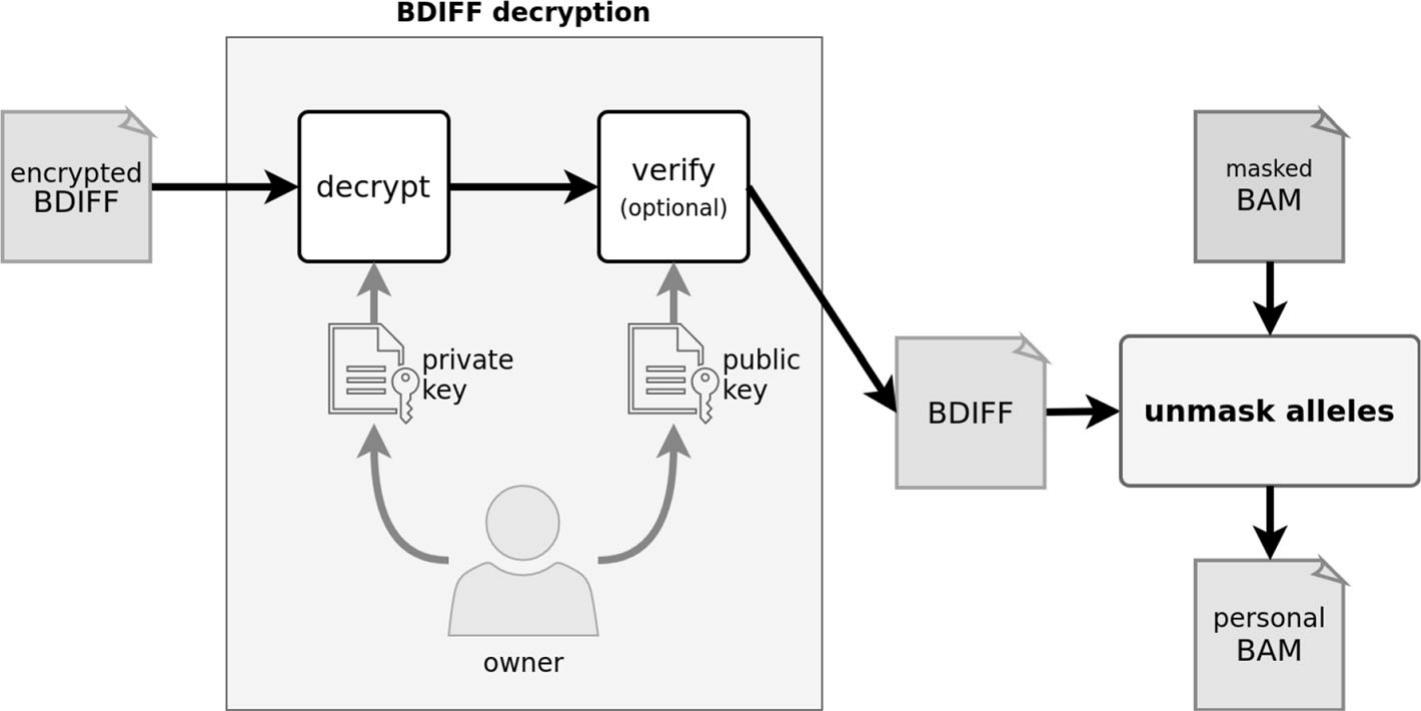
Workflow of the unmasking method, where the BDIFF file is decrypted and used to unmask the masked BAM file to restore the personal BAM file

**Fig. 10:**
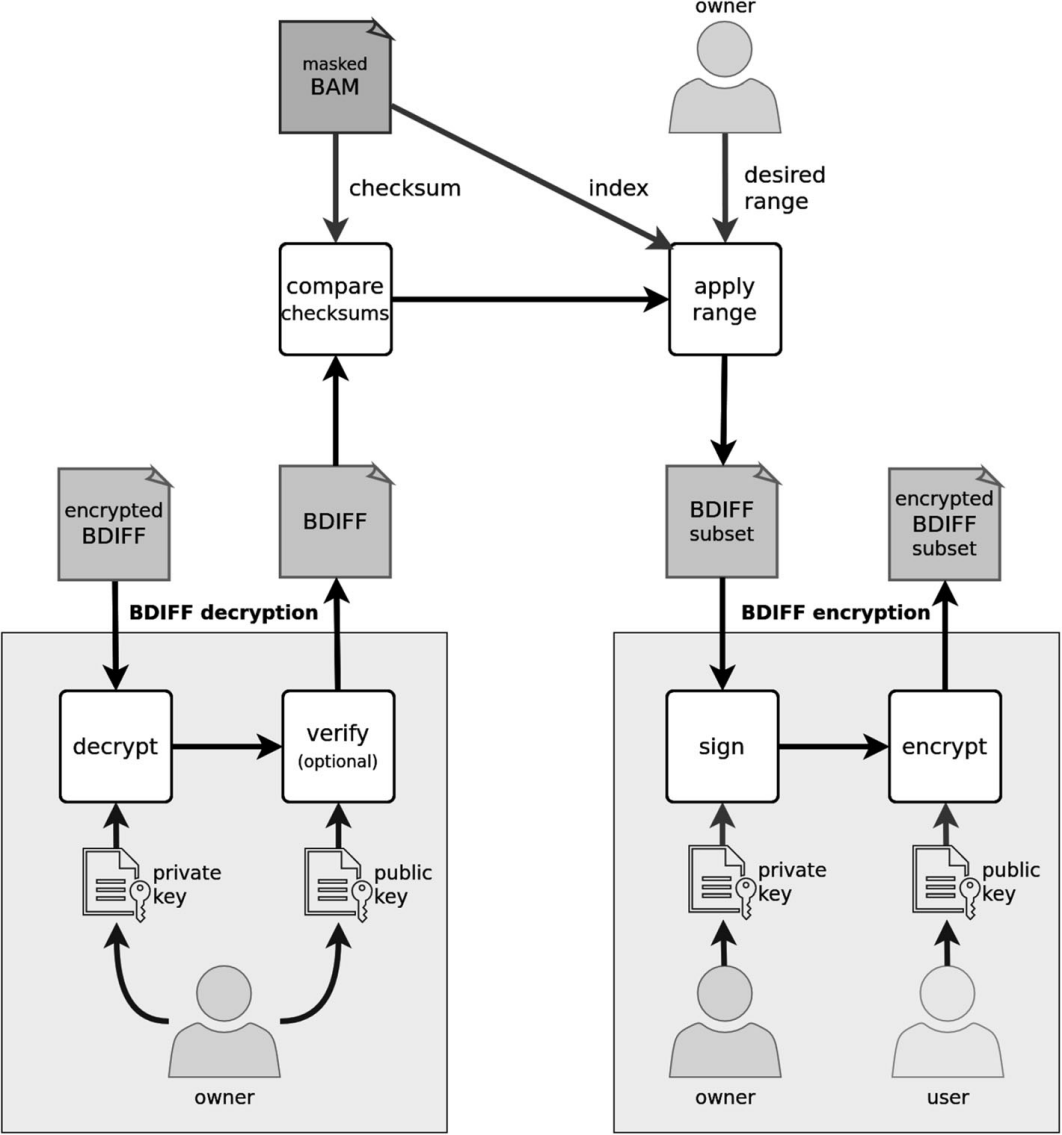
Workflow of the sharing method showing decryption of BDIFF and encryption of its subrange intended for a specific user

The unmasking method (Fig. 9) represents a partially reversed masking method. The file with masked alleles is decrypted with the associated private key and is processed simultaneously with masked alignments back into personal alignments. The dissemination (Fig. 10) method re-encrypts the file with masked alleles in an arbitrary range, making the associated subset of alleles accessible to a specific user. Firstly, the file with masked alleles is decrypted by the associated private key. Secondly, a subset of masked alleles is selected, and lastly, the selected masked alleles are encrypted as a new file with the public key of a specific user.

### Masking of alleles

A sequenced genomic position is typically covered by multiple alignments, which may carry different alleles due to heterozygosity, sequencing, or alignment errors. Both personal alleles are equally likely to be represented in the alignments, albeit their mutual ratio can substantially vary for any given position. Therefore, each genomic position with a population variant is described as a list of alleles, and a personal pair of alleles is determined as the two most represented ones. In detail, an allele is considered personal if it constitutes a sufficiently large portion of alignments covering the position of a variant [22]. If only one such allele exists, the position is evaluated as homozygous, and two identical alleles are assigned to the position. If two different alleles with sufficient representation exist, the position is considered heterozygous, and two different alleles are assigned to the position. If there are more than two sufficiently represented alleles, the methodology skips the variant position.

**Fig. 11:**
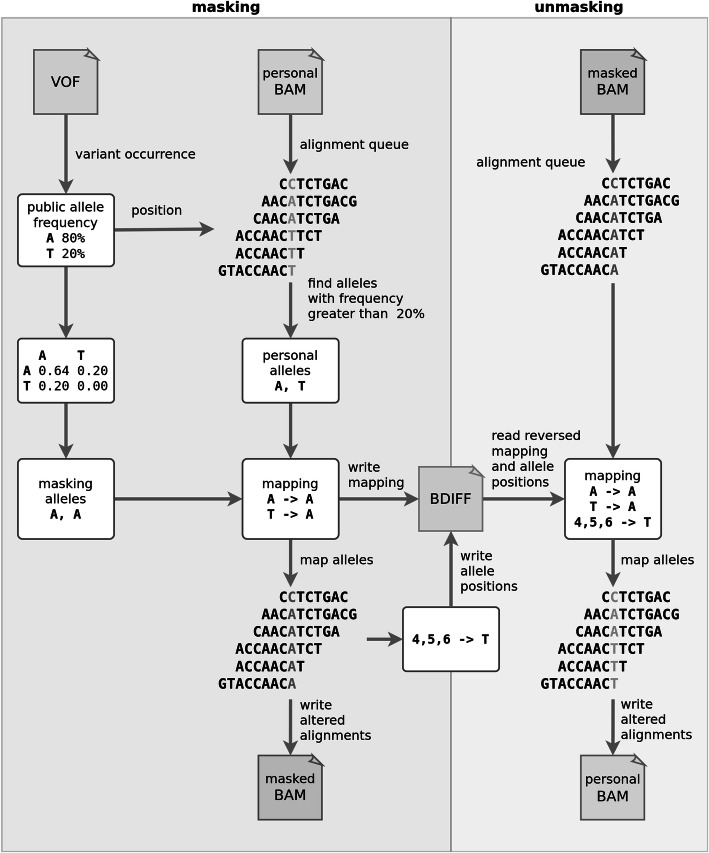
Flow of masking and unmasking alleles at a single variant position within covering alignments. The masking is represented as “mask alleles” in Fig. 8, and the unmasking is represented as “unmask alleles” in Fig. 9

The process of masking and unmasking alleles per any given position has several steps (Fig. 11). Each allele from the pair of masking alleles is selected randomly from the multinomial distribution of population alleles. The masking pair of alleles acts as a replacement for the pair of personal alleles assigned previously. Moreover, the zygosity at the masked position can change from homozygous to heterozygous and vice versa (If a reference allele replaces both alternative personal alleles, a variant cannot be detected in masked alignments and it therefore becomes masked. Conversely, if an alternative allele replaces either of the personal reference alleles, a new variant can be called at this position in masked alignments and it thus becomes introduced. All personal alleles within the alignments covering a particular variant are replaced by masking alleles. However, personal alleles may be replaced by the same pair of masking alleles, which is the most common case - a pair of reference alleles is mapped to itself. The remaining alleles found within the alignments are considered to be sequencing or alignment errors. These alleles can still be replaced by other than masking alleles. We elaborate more on the aspects of the masking process in Additional file 1; Section 3.

### Unmasking of alleles

All alleles within masked alignments, or their specific subsets, can be unmasked by the BDIFF file, containing the replaced personal alleles and deleted qualities. This operation transforms masked alignments to original alignments. User has to provide masked alignments and an associated encrypted BDIFF file along with the RSA private key whose public counterpart was used in the BDIFF encryption. The decryption of unmapped reads is handled separately, and the user can choose whether to decrypt them or not.

The first step of the unmasking method is the decryption of the encrypted BDIFF file (Additional file 1; Section 2; Fig. 1). The algorithm reads the encrypted AES key and the file signature from the beginning of the file. The AES key is decrypted with a provided private key and then used to decrypt the actual encrypted BDIFF file. The decrypted file is verified with a public key against its signature to prove its origin.

### Sharing of alleles

The holder of the private key that was used to encrypt the BDIFF file can share the alleles described by the BDIFF file and associated masked alignments by re-encrypting the BDIFF file in the desired genomic range. The BDIFF file is first decrypted by the private key and then encrypted by the public key of another user who can decrypt the file later. If a subrange of the effective range for re-encryption is provided, only records inside or intersecting this range are considered, and this range becomes the effective range of the new BDIFF file. The re-encryption process can be repeated with different combinations of genomic ranges and public keys, producing different accesses for individual users. In addition, the decrypted BDIFF file can be verified with the holder’s public key by comparing the checksum of masked alignments to the checksum stored in the encrypted BDIFF file header. This ensures that the BDIFF file belongs to the masked alignments and that they were not modified.

### Validation

To validate the Varlock, we collected a set of 33 clinical exomes from the central European population. The DNA samples were sequenced on Illumina platform following enrichment and library preparation using TruSight One clinical exome sequencing panel following the manufacturer’s instructions. Next, we called variants on each exome with a fine-tuned variant calling pipeline comprising the BWA-MEM alignment mapper algorithm [18] and DeepVariant caller [24].The pipeline produced 33 BAM files mapped to the GRCh38 reference genome and the same number of corresponding VCF files. Finally, we masked each BAM file with the Varlock and subsequently called variants on these masked BAM files thus producing the same number of VCF files.

In addition, we used the third release of the 1000 Genomes Project as the source of samples for the second PCA analysis [1]. We selected the first five exome samples from the non-Finnish European (Toscani) population and the first five exome samples from the African (African Caribbean) population. We provide the list of these samples in the Additional file 2.

As the source of population variants, we used The Genome Aggregation Database version 3 (gnomAD v3) [15] mapped to the GRCh38 reference genome, which spans 71,702 genomes from unrelated individuals of various ethnicities. We downloaded the database in the form of a single VCF file, selected the passing SNVs within ranges of Trusight One clinical exome panel, and merged duplicate variant positions as multiallelic. Finally, we converted the file to VOF format intended for masking.

To validate masking of pathogenic variation we annotated the VCF files in our dataset with the Ensembl Variant Effect Predictor using the cache version 101 [19] providing gene annotations in order to filter relevant variants, which were manually examined later.

## Supplementary Information

**Additional file 1.** Additional results and methods.

**Additional file 2.** List of selected samples from the 1000 Genomes Project.

## Data Availability

The source code of Varlock is available in the GitHub repository under Creative Commons Attribution-NonCommercial-ShareAlike 4.0 International Public License and includes unit tests for individual methods (https://github.com/rtcz/varlock). The source code is archived in Zenodo repository (https://zenodo.org/record/4543247, doi:10.5281/zenodo.4543247). The tool is coded in Python 3 and is platform independent. We validated the method with the population data from The Genome Aggregation Database version 3 (gnomAD v3) (https://gnomad.broadinstitute.org/downloads), To compare the effect of the masking method between two distinct populations, we used samples publicly available from the data portal of 1000 Genomes Project (https://www.internationalgenome.org/data-portal/sample). The samples can be searched with the accession numbers provided in Additional file 2 and downloaded from this portal. We annotated the VCF files with additional data with the tool Ensembl Variant Effect Predictor version 101, which downloaded annotation data automatically. The clinical exomes dataset used to evaluate Varlock is not publicly available due to personal data protection but is available from the corresponding author on a reasonable request.

## Abbreviations

ACMG: American College of Medical Genetics
AES: Advanced Encryption Standard; a specification of a symmetric encryption scheme
BAM: Binary Alignment Map; a file format specification for storing genomic reads aligned to reference sequence
BDIFF: File format introduced in Varlock; stores differences between masked and personal BAM; detailed in Additional file 1; Section 2; Table 3–4
CRAM: Compressed Columnar File Format; an alternative to BAM
PCA: Principal Component Analysis
RSA: Rivest-Shamir-Adleman cryptosystem; an asymmetric encryption scheme using a public and a private key
SNV: Single Nucleotide Variant
VCF: Variant Call Format; a file format specification for storing genomic variations
VOF: File format introduced in Varlock; stores population allele frequencies

## References

[1] Auton A, Brooks LD, Durbin RM, Garrison EP, Kang HM, 1000 Genomes Project Consortium (2015). A global reference for human genetic variation. Nature.

[2] Ashley EA (2016). Towards precision medicine. Nat Rev Genet.

[3] Ayday E, De Cristofaro, Hubaux J-P, Tsudik G. The chills and thrills of whole genome sequencing. Computer. 2013a. 10.1109/mc.2013.333.

[4] Ayday E, Raisaro JL, Hubaux J-P, Rougemont J. Protecting and evaluating genomic privacy in medical tests and personalized medicine. In: Proceedings of the 12th ACM workshop on workshop on privacy in the electronic society, 95–106: ACM; 2013b.

[5] Ayday E, Raisaro JL, Hengartner U, Molyneaux A, Hubaux J-P (2014). Privacy-preserving processing of raw genomic data. Data privacy management and autonomous spontaneous security, edited by Joaquin Garcia-Alfaro, Georgios Lioudakis, Nora Cuppens-Boulahia, Simon Foley, and William M. Fitzgerald, 8247:133–47. Lecture notes in computer science.

[6] Budis J, Gazdarica J, Radvanszky J, Harsanyova M, Gazdaricova I, Strieskova L (2019). Non-invasive prenatal testing as a valuable source of population specific allelic frequencies. J Biotechnol.

[7] Carter AB (2019). Considerations for genomic data privacy and security when working in the cloud. J Mol Diagnost.

[8] Cogo VV, Bessani A, Couto FM, Verissimo P. A high-throughput method to detect privacy-sensitive human genomic data. In: Proceedings of the 14th ACM workshop on privacy in the electronic society, 101–10: ACM; 2015.

[9] Decouchant J, Fernandes M, Voelp M, Couto FM, Esteves-Verissimo P. Accurate filtering of privacy-sensitive information in raw genomic data; 2018. 10.1101/292185. 10.1016/j.jbi.2018.04.006 29660494

[10] Erlich Y, Narayanan A (2014). Routes for breaching and protecting genetic privacy. Nat Rev Genet.

[11] Frizzo-Barker J, Chow-White PA, Charters A, Ha D (2016). Genomic big data and privacy: challenges and opportunities for precision medicine. Comput Support Coop Work.

[12] Huang Z, Ayday E, Lin H, Aiyar RS, Molyneaux A, Xu Z (2016). A privacy-preserving solution for compressed storage and selective retrieval of genomic data. Genome Res.

[13] Jagadeesh KA, Wu DJ, Birgmeier JA, Boneh D, Bejerano G (2017). Deriving genomic diagnoses without revealing patient genomes. Science.

[14] Kalia SS, Adelman K, Bale SJ, Chung WK, Eng C, Evans JP (2017). Recommendations for reporting of secondary findings in clinical exome and genome sequencing, 2016 update (ACMG SF v2.0): a policy statement of the American College of Medical Genetics and Genomics. Genet Med.

[15] Karczewski KJ, Francioli LC, Tiao G, Cummings BB, Alföldi J, Wang Q (2020). The mutational constraint spectrum quantified from variation in 141,456 humans. Nature.

[16] Kubiritova Z, Gyuraszova M, Nagyova E, Hyblova M, Harsanyova M, Budis J (2019). On the critical evaluation and confirmation of germline sequence variants identified using massively parallel sequencing. J Biotechnol.

[17] Lauter K, López-Alt A, Naehrig M. Private computation on encrypted genomic data. In: Progress in cryptology - LATINCRYPT 2014, 3–27: Springer International Publishing; 2015.

[18] Li H, Durbin R. Fast and accurate short read alignment with burrows–wheeler transform. Bioinformatics. 2009;25(14):1754–60. 10.1093/bioinformatics/btp324 PMC2705234 19451168

[19] McLaren W, Gil L, Hunt SE, Riat HS, Ritchie GRS, Thormann A, et al. The ensembl variant effect predictor. Genome Biol. 2016;17(1):122. 10.1186/s13059-016-0974-4 PMC4893825 27268795

[20] Minarik G, Repiska G, Hyblova M, Nagyova E, Soltys K, Budis J (2015). Utilization of Benchtop next generation sequencing platforms ion torrent PGM and MiSeq in noninvasive prenatal testing for chromosome 21 trisomy and testing of impact of in Silico and physical size selection on its analytical performance. PLoS One.

[21] Mohammed Yakubu A, Chen Y-PP (2020). Ensuring privacy and security of genomic data and functionalities. Brief Bioinform.

[22] Muzzey D, Evans EA, Lieber C (2015). Understanding the basics of NGS: from mechanism to variant calling. Curr Genet Med Rep.

[23] Naveed M, Ayday E, Clayton EW, Fellay J, Gunter CA, Hubaux J-P, et al. Privacy in the genomic era. ACM Comput Surv. 2015;48(1). 10.1145/2767007. 10.1145/2767007 PMC4666540 26640318

[24] Poplin R, Chang P-C, Alexander D, Schwartz S, Colthurst T, Alexander K (2018). A universal SNP and small-indel variant caller using deep neural networks. Nat Biotechnol.

[25] Pös O, Budis J, Kubiritova Z, Kucharik M, Duris F, Radvanszky J, et al. Identification of structural variation from NGS-based non-invasive prenatal testing. Int J Mol Sci. 2019a;20(18). 10.3390/ijms20184403. 10.3390/ijms20184403 PMC6769840 31500242

[26] Pös O, Budiš J, Szemes T. Recent trends in prenatal genetic screening and testing. F1000Research. 2019b;8(May). 10.12688/f1000research.16837.1. 10.12688/f1000research.16837.1 PMC6545823 31214330

[27] Purcell S, Neale B, Todd-Brown K, Thomas L, Mar D (2007). PLINK: a tool set for whole-genome association and population-based linkage analyses. Am J Hum Genet.

[28] Richards S, Aziz N, Bale S, Bick D, Das S, Gastier-Foster J (2015). Standards and guidelines for the interpretation of sequence variants: a joint consensus recommendation of the American College of Medical Genetics and Genomics and the Association for Molecular Pathology. Genet Med.

[29] Sariyar M, Suhr S, Schlünder I (2017). How sensitive is genetic data?. Biopreserv Biobank.

[30] Schwab AP, Luu HS, Wang J, Park JY (2018). Genomic privacy. Clin Chem.

[31] Shabani M, Marelli L. Re-identifiability of genomic data and the GDPR: assessing the re-identifiability of genomic data in light of the EU general data protection regulation. EMBO Rep. 2019;20(6). 10.15252/embr.201948316. 10.15252/embr.201948316 PMC6549023 31126909

[32] Shen H, Ma J (2017). Privacy challenges of genomic big data. Adv Exp Med Biol.

[33] Sousa JS, Lefebvre C, Huang Z, Raisaro JL, Aguilar-Melchor C, Killijian M-O (2017). Efficient and secure outsourcing of genomic data storage. BMC Med Genom.

