# Supplementary material for: Privacy-preserving storage of sequenced genomic data
Source: BMC Genomics. 2021 Oct 2;22:712. doi: 10.1186/s12864-021-07996-2 (PMC8487550; doi:10.1186/s12864-021-07996-2)
Supplement: Supplementary file 1 — Additional file 1. Additional results and methods. [file 12864_2021_7996_MOESM1_ESM.pdf]

Additional file 1

1 Variant occurrence format

VOF is a compact file format storing the numbers of SNV or INDEL alleles for specific genomic positions. These numbers represent the incidence of alleles within a population. Format stores two similar types of records sorted by genomic position, one for SNV alleles and the second for INDEL alleles, and has four distinct fields displayed in Table 1.

**position**	Position of the variant within the genome.
**type**	SNV or INDEL
**reference index**	In the case of an SNV, it is the index of the reference allele in A, T, G, C list. In case of an INDEL, it is the index of the listed allele.
**allele counts**	The numbers of observed alleles in a population. In an SNV record, allele numbers are corresponding to the list of A, T, G, C alleles respectively. INDEL alleles are listed explicitly, and the occurrence count is assigned to each of them.

Table 1: *Data record stored for each variant in the VOF file format.*

**position**	**type**	**reference index**	**allele counts**
11042	0	2	0, 89, 1, 10
11191	1	1	TC: 5, TCA: 95

Table 2: *An example of records in a VOF file.*

2 BDIFF format

All SNV and INDEL alleles replaced in personal mapped reads are stored in a BDIFF format. The BDIFF file format provides a header for storing metadata required in the masking process and a file index enabling fast seeking of genomic positions. BDIFF records are sorted by the genomic position. A single BDIFF record stores the difference between original and masked allele in four fields displayed in Table 3.

**position**	Position of the variant within the genome.
**type**	SNV or INDEL.
**reference index**	In the case of an SNV, it is the index of the reference allele in A, T, G, C list. In case of an INDEL, it is the index of the listed allele.
**allele mapping**	Listed SNV alleles correspond to A, T, G, C bases respectively. Each listed INDEL allele has an index pointing to a target allele from the list.

Table 3: *Data record stored for each variant in the BDIFF file format.*

**position**	**type**	**reference index**	**allele mapping**
11032	0	2	G, A, T, A
11038	1	0	GCG: 1, G: 0

Table 4: *An example of records in a BDIFF file.*

When two different personal alleles are replaced by two identical masking alleles as part of the masking process described later (masking from heterozygous to homozygous position), information necessary to reverse this operation is lost. It is impossible to infer which particular alignment with the masked allele was the carrier of which personal allele from the original pair. This problem is resolved by keeping one of the replaced personal alleles as a part of a BDIFF record together with the list of identifiers of alignments associated with this allele. The other replaced personal allele is mapped to a masking allele as usual.

In addition, the BDIFF file needs to keep deleted base qualities associated with replaced alleles. Base qualities are deleted only when the longer allele is replaced with shorter allele, which is a case of INDEL masking. Deleted quality sequences are stored in another field of INDEL record as a list sorted by the genomic position of the corresponding alignment.

2.1 BDIFF encryption and storage

The checksum of the mapped reads and checksum of the VOF file is stored along with masked mapped reads for later verification (Figure 1). After masked mapped reads are complete, their checksum is added to the header of the encrypted BDIFF file.

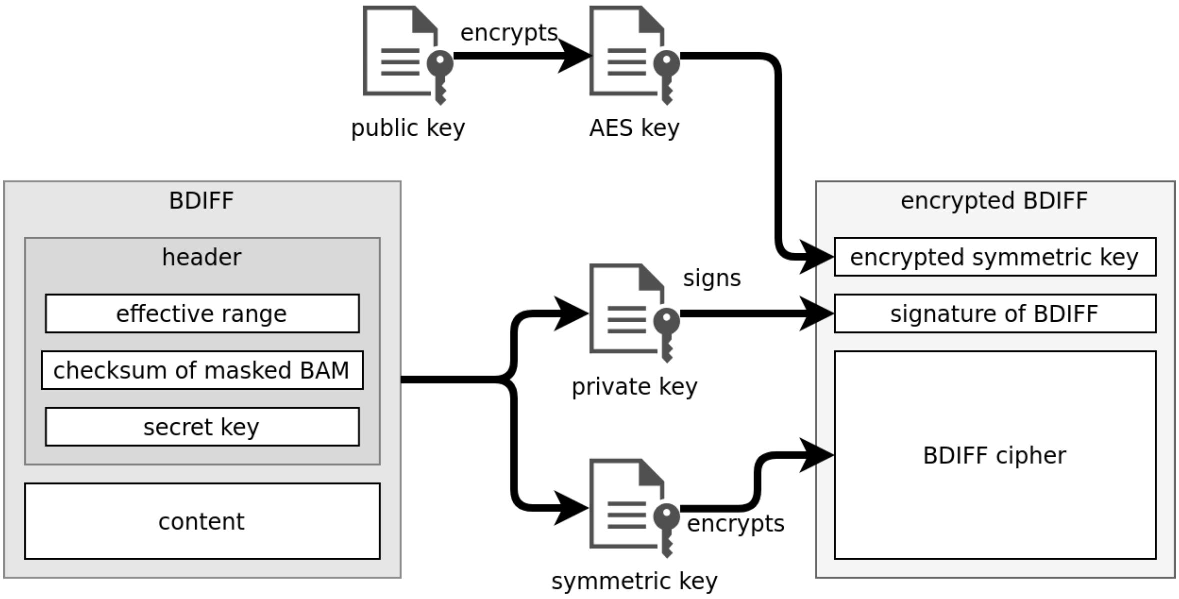

Figure 1: *Conversion process from in-memory BDIFF to encrypted BDIFF file stored on disk.*

BDIFF file header contains the exact range that the masking covers - effective range. It is necessary because a BDIFF file does not have to contain records with genomic positions exactly at the start and the end of a specific range. By default, the effective range covers the whole genome. An owner of a BDIFF file can specify a subrange of an effective range to produce a new smaller BDIFF. This process is called dissemination and is explained later. Effective range is always greater or equal to a range defined by the first and last BDIFF record. The secret key for encryption of unmapped reads and checksum of masked mapped reads are also stored within the BDIFF header.

BDIFF contains all of the information necessary to unmask personal alleles within masked mapped reads, hence it is never stored as plain text. Content of the BDIFF file is encrypted using AES encryption with a randomly generated key. The AES key itself is encrypted by an RSA public key, provided by the user, and stored as a part of the encrypted BDIFF file. In this way, access to the personal alleles is restricted to the owner of the private key paired with the public key used for encryption. Finally, the plain BDIFF file is signed with a provided private key. The signature is stored as a part of an encrypted BDIFF file and verified at the start of a decryption process using a public key paired with a signing private key.

3 Masking

If the masking process alters any of the alignments covering the position of a variant, the mapping from personal alleles to masking alleles is stored in the BDIFF format. After all positions of the variants on an alignment, described by population allele frequencies, and

all preceding alignments are treated, the alignment is stored as a masked mapped read. When all positions of variants are processed, remaining alignments, although unchanged, are stored as well. Since potential alleles in unmapped reads are not masked in the process, they are completely encrypted by random nucleotides. In addition, random masking can be further employed to increase personal privacy by obfuscation. We describe these two methods hereinafter.

3.1 SNVs

An allele of a single nucleotide variant (SNV) is one of four DNA bases; therefore, the number of possible SNV alleles is always four. Accordingly, the probability matrix of SNV allele pairs has size 4x4, where the probability of each pair is the product of their population frequencies. Unknown allele (typically denoted as *N* in sequence files) is mapped to itself; thus, it is always preserved.

3.2 INDELs

In case of an insertion or a deletion (INDEL), the number of possible alleles depends on the number of different alleles found within alignments at the position of a variant. In order to find an actual INDEL allele within a particular alignment, population alleles defined by VOF records are iterated from the longest to the shortest one. In each iteration, the CIGAR string of the current allele is inferred from the difference between its length and the length of reference allele. Length of the shorter allele from the pair is considered to be a number of CIGAR match operations. The difference between the two lengths is either positive or negative, denoting the number of CIGAR insertions or the number of CIGAR deletions, respectively. The computed CIGAR string of the current allele is compared with the corresponding portion of CIGAR string describing the alignment. Likewise, the nucleotide sequence of the allele is compared with the corresponding subsequence of the alignment. If both sequences and CIGAR strings match, the actual allele is found, and iteration is stopped.

The probability matrix of INDEL allele pairs is created from the found alleles, where probabilities are determined as in the SNV case. A personal allele is replaced with a masking allele affecting both nucleotide sequence and CIGAR string. The alignments without any detected personal allele remain unchanged.

3.3 Unmapped reads

Unmapped reads are encrypted completely using stream cypher encryption which produces a cypher with the same size as the input. At first, a secret key is randomly generated for all

unmapped reads. This key is stored within the BDIFF file header. When an unmapped read is found, its template name and the secret key are hashed by SHA algorithm producing 512 bits long hash. The hash is then used to encrypt the sequence of the read. Every two bits of the hash are used to encrypt one DNA base, also encoded by 2 bits, from the input sequence using a simple XOR operation. Consequently, the key size is enough to encrypt a sequence of 256 bases uniquely. If the sequence is longer, the key is repeated. Unknown bases, represented by letter N, are skipped in the encryption.

3.4 Random masking

The provided VOF file and the masked mapped reads are considered public; therefore, everybody can tell which positions on a genome could be masked. As a consequence, rare variants not covered by the VOF file can be still abused by an adversary to infer personal data. This vulnerability is mitigated by the introduction of random, artificial SNV alleles into masked mapped reads by generating additional random VOF records before the masking process. Each generated VOF record has a random genomic position and contains allele counts representing approximate ratios of alleles in the human genome. Both generated and file contained VOF records are iterated together and processed in the same way. As a result, generated VOF records have a chance to mask or introduce novel variants in the same way as a population based record. The number of new variants should be high enough to disallow attacks in-between the variants from the VOF file. On the other hand, the size of BDIFF file and time cost of all operations linearly increases with the increasing number of variants.

3.5 CIGAR string and sequencing quality

Mapped reads do not contain only nucleotide sequences, but also other sensitive data that we process. When making a modification to alignment, the CIGAR string is modified accordingly; otherwise, it would be easy to guess the nature of an original alignment. Moreover, mapped alignment typically contains sequence qualities that express confidence in each base. While a masking SNV allele does not change the length of the alignment, a masking INDEL allele often does, so it is necessary to adjust the length of a sequencing quality string to match the altered alignment. If the masked alignment is longer than the original one, the masking method provides artificial qualities to fill the gap. On the other hand, if the masked alignment is shorter than the original one, sequencing qualities are deleted and stored within a BDIFF file to keep the masking method reversible.

3.6 Masking cases

Both personal and masking pair can be either homozygous or heterozygous, leading to one of the following cases:

Homozygous to homozygous: Two identical masking alleles replace two identical personal alleles. Most often, two reference alleles are replaced by the same two alleles, since the reference allele is typically the most common one in both personal mapped reads and population allele frequencies. If pairs are identical, no actual masking occurs. Possible outcome: masked variant, introduced variant, replaced variant, none.

Heterozygous to homozygous: Two identical masking alleles replace two different personal alleles. Reference and alternative alleles are often replaced by two reference alleles, which results in masking of a personal variant. Possible outcome: masked variant, replaced variant.

Homozygous to heterozygous: Two different masking alleles replace two identical personal alleles. If an alternative allele replaces either reference allele, a new variant emerges. Possible outcome: introduced variant, replaced variant.

Heterozygous to heterozygous: Two different masking alleles replace another two different personal alleles. Personal and masking pairs of these alleles are often identical, so no actual masking occurs. If only one personal allele is identical to a masking allele, the other personal allele is masked with the remaining masking allele. In this case, the position of a variant is not masked since the alternative allele is replaced by another alternative allele. Possible outcome: replaced variant, none.

4 Masking effect

We examined the masking effect on personal alleles on non-Finnish European (NFE), African (AFR), and South Asian (SAS) populations separately with corresponding population allele frequencies (Figure 2-4). For this purpose we selected samples from the third phase of the 1000 Genomes Project mapped to the GRCh38 reference genome (https://www.internationalgenome.org/data-portal/sample). We provide the list of these samples in the Additional file 2.

Masked samples stay within the same population space and are shuffled within a main cluster. In the case of the SAS population (Figure 4), there is only a negligible shift from personal to masked samples, since its size is 1,526 genomes only. As can bee seen this number is not large enough for an effective masking and the masking effectiveness depends on the comprehensiveness of population allele frequencies. In comparison, NFE and AFR populations have 32,299 and 21,042 genomes respectively.

Moreover, we masked all the three populations of samples with all the The Genome Aggregation Database version 3 (GnomAD v3) population allele frequencies (https://gnomad.broadinstitute.org/downloads), causing shift towards a common area (Figure 5). However, most masked samples do not overlap with masked samples from a different population, hence this approach can not be used for masking the population of a sample origin with current size of the gnomAD population allele frequencies.

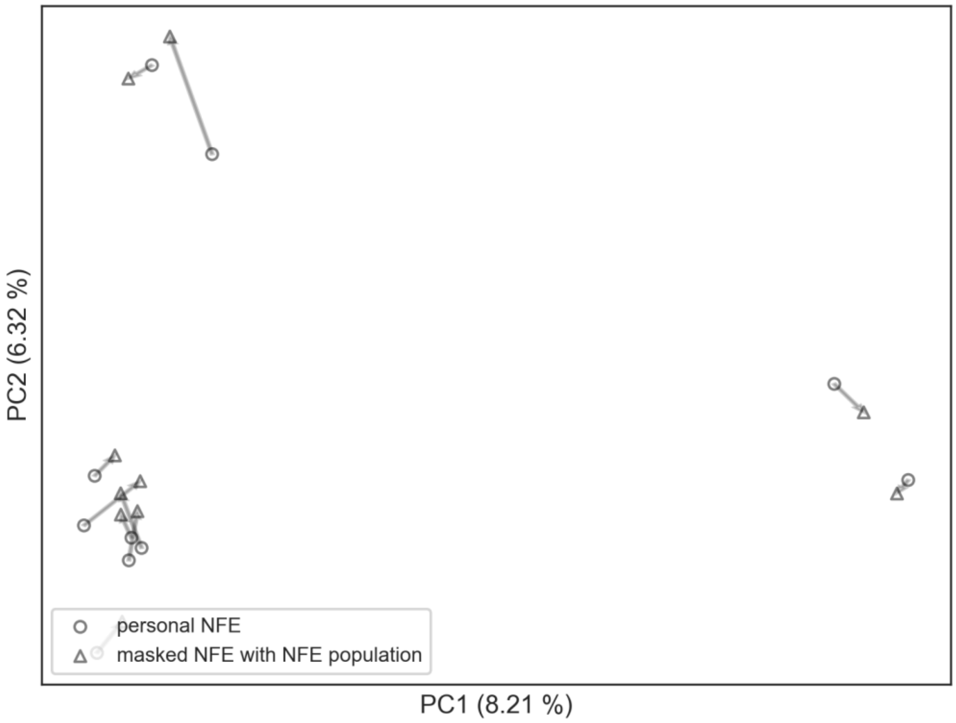

Figure 2: *The PCA of ten personal and corresponding masked samples from non-Finnish European (NFE) population masked with NFE population frequencies.*

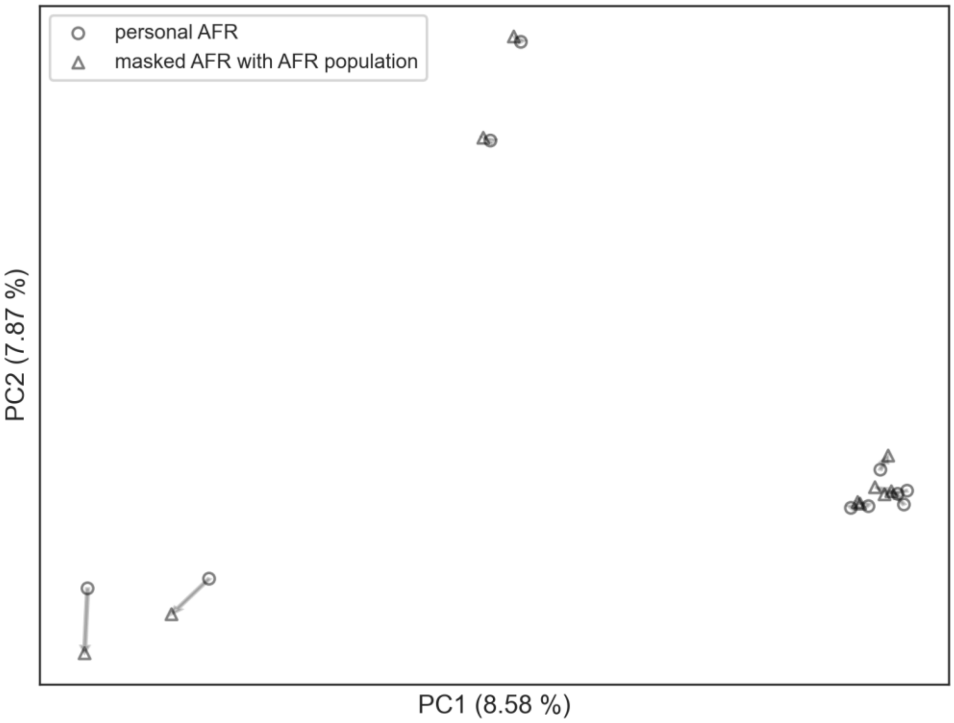

Figure 3: *The PCA of ten personal and corresponding masked samples from African (AFR) population masked with AFR population frequencies.*

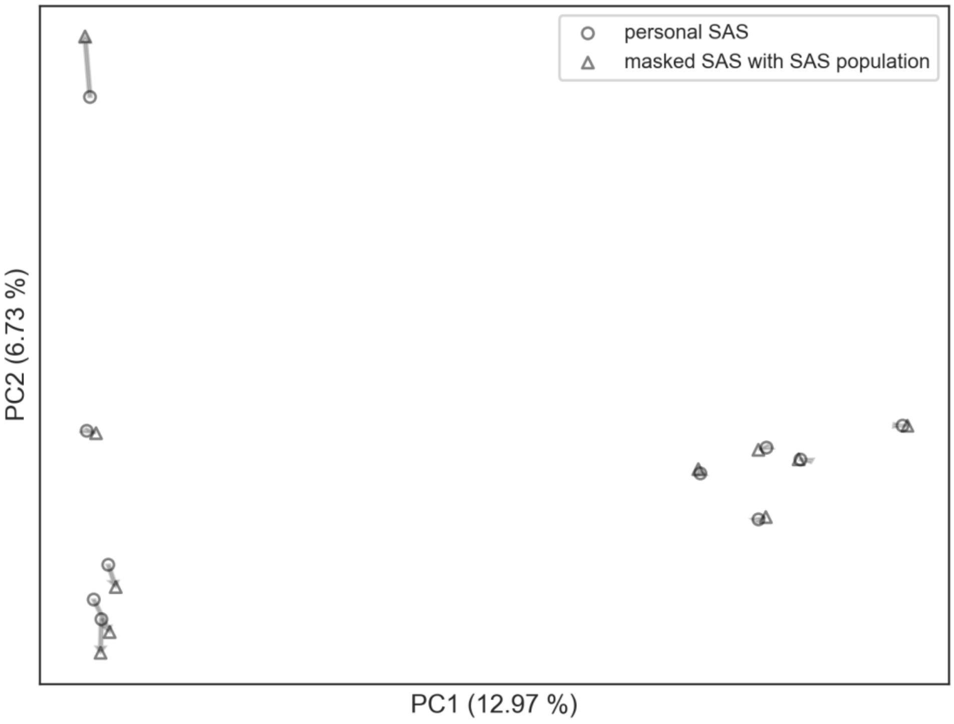

Figure 4: *The PCA of ten personal and corresponding masked samples from South Asian (SAS) population masked with SAS population frequencies.*

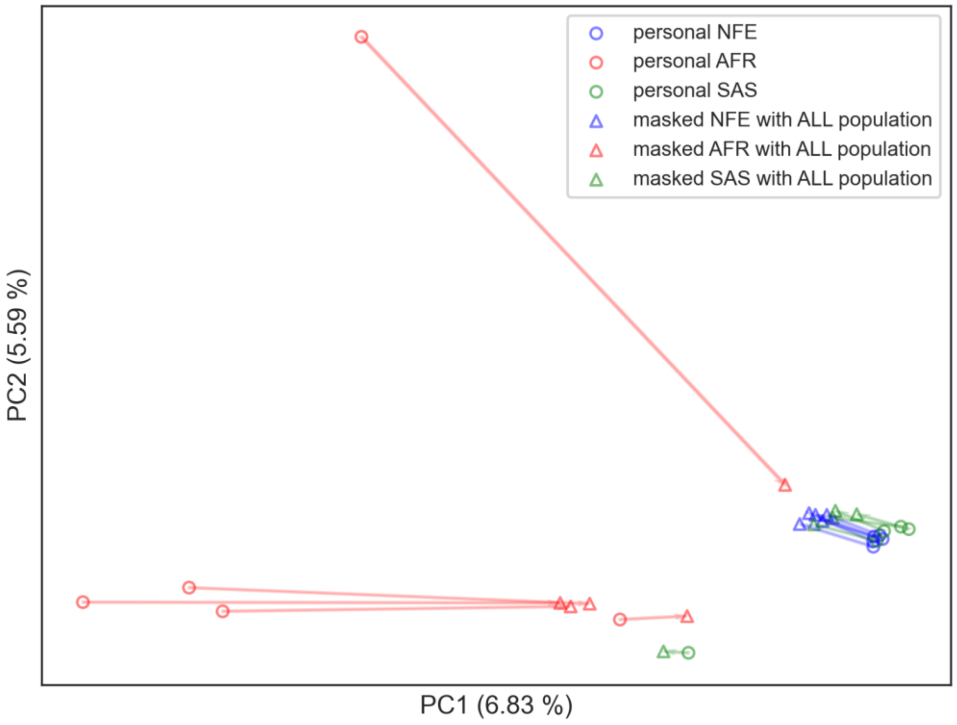

Figure 5: *The PCA of five non-Finnish European (NFE), five African (AFR), and five South Asian (SAS) samples, all masked with all gnomAD population allele frequencies (ALL).*
